# Supplementary material for: Borosilicate Glass-Ceramics Containing Zirconolite and Powellite for RE- and Mo-Rich Nuclear Waste Immobilization
Source: Materials (Basel). 2021 Oct 1;14(19):5747. doi: 10.3390/ma14195747 (PMC8510122; doi:10.3390/ma14195747)
Supplement: Supplementary file 1 [file materials-14-05747-s001.zip › materials-1357084-supplementary.pdf]

materials

MDPI

Supplementary Material

Borosilicate Glass-Ceramics Containing Zirconolite and Powellite for RE- and Mo- Rich Nuclear Waste Immobilization

Wei Wan [1], Yongchang Zhu [2,*], Xingquan Zhang [3,*], Debo Yang [2], Yonglin Huo [1], Chong Xu [1], Hongfu Yu [1], Jian Zhao [1], Jichuan Huo [1] and Baojian Meng [2]

[1] Fundamental Science on Nuclear Wastes and Environmental Safety Laboratory, Southwest University of Science and Technology, Mianyang 621010, China; wanwei992695714@163.com (W.W.); hyl20201016@163.com (Y.H.); yjcbma@163.com (C.X.); yuhongfu2018@mails.swust.edu.cn (H.Y.); z18210433040@163.com (J.Z.); huojichuan@swust.edu.cn (J.H.)
[2] China Building Materials Academy, Beijing 100024, China; yangdebo19770904@126.com (D.Y.); Baojian_meng@163.com (B.M.)
[3] School of Materials Science and Engineering, Southwest University of Science and Technology, Mianyang 621010, China
* Correspondence: zhuyongchang@cbma.com.cn (Y.Z.); zhangxingquan@swust.edu.cn (X.Z.); Tel./Fax: +86-65794978 (Y.Z.); +86-816-6089508 (X.Z.)

Table S1. Structural Rietveld refinement results of the Nd-doped zirconolite phase for CTZ-40 sample.

Site	x	y	z	Mult	Occupancy	Uiso (Å^2)
CTZ-40: $Ca_{0.78}Nd_{0.22}ZrTi_{1.78}Al_{0.22}O_7$,space group *C*12/*c*1; Z=8;a=12.7909(1) Å, b=7.3946(4) Å, c=11.6172(1) Å; α=90.000(0) °, β=100.900(6) °, γ=90.000(0) °;V=1078.97(8) Å^3						
Ca(1)	0.3730	0.1230	0.4940	8	0.74	0.0268
Nd(1)	0.3810	0.1280	0.4939	8	0.22	0.0700
Zr(1)	0.3590	0.1250	0.4959	8	0.04	0.0220
Ca(2)	0.1212	0.1222	0.9744	8	0.04	0.0250
Zr(2)	0.1222	0.1222	0.9744	8	0.88	0.0240
Ti(1)	0.1232	0.1222	0.9744	8	0.08	0.0241
Zr(3)	0.2397	0.1120	0.7377	8	0.03	0.0190
Ti(2)	0.2497	0.1220	0.7467	8	0.97	0.0270
Zr(4)	0.04740	0.0560	0.2500	8	0.05	0.0250
Ti(3)	0.4740	0.0560	0.2500	8	0.23	0.0401
Al(1)	0.4770	0.0580	0.2600	8	0.22	0.0250
Ti(4)	0.0000	0.1270	0.2500	4	1.00	0.0341
O(1)	0.3110	0.1370	0.2750	8	1.00	0.0211
O(2)	0.4700	0.1440	0.1020	8	1.00	0.0199
O(3)	0.1960	0.0820	0.5740	8	1.00	0.0250
O(4)	0.4040	0.1750	0.7200	8	1.00	0.0220
O(5)	0.7030	0.1680	0.5910	8	1.00	0.0231
O(6)	0.0010	0.1110	0.4140	8	1.00	0.0180
O(7)	0.1190	0.0540	0.7870	8	1.00	0.0182
